# Supplementary material for: Protection against overfeeding-induced weight gain is preserved in obesity but does not require FGF21 or MC4R
Source: Nat Commun. 2024 Feb 8;15:1192. doi: 10.1038/s41467-024-45223-0 (PMC10853283; doi:10.1038/s41467-024-45223-0)
Supplement: Supplementary file 1 — Supplementary Information [file 41467_2024_45223_MOESM1_ESM.pdf]

Protection against overfeeding-induced weight gain is preserved in obesity but does not require FGF21 or MC4R

Supplementary information file

Supplementary Figures

Supplementary Figure 1

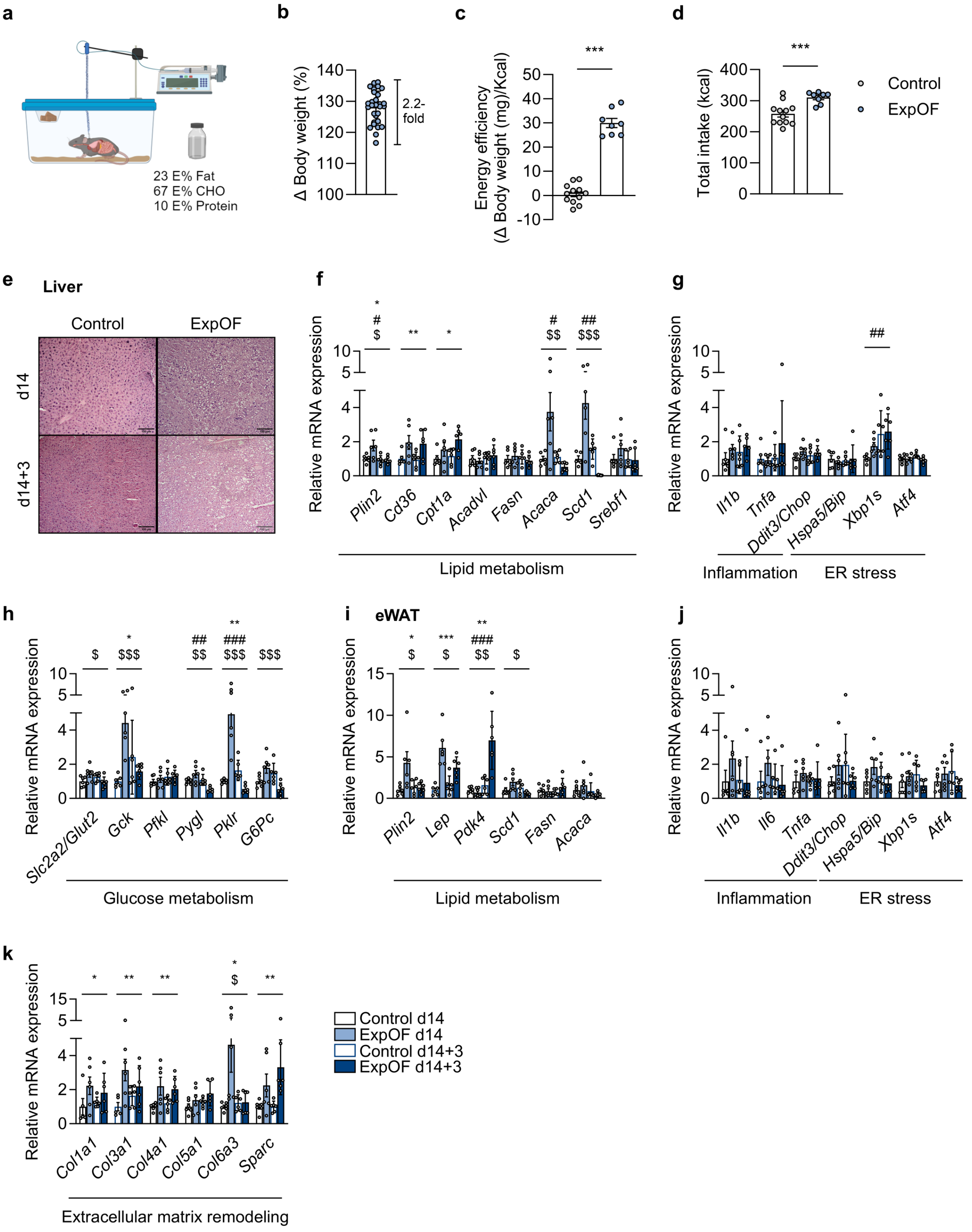

Supplementary Figure 1. Effects of overfeeding on lean mice

a) Schematic overview of the experimental overfeeding (ExpOF) setup and macronutrient composition of infused liquid diet. Created with BioRender.com **b**) Individual weight gain (%) of all WT chow mice subjected to experimental overfeeding was used to calculate fold-change between highest and lowest gainers in the study. **c**) Energy efficiency (mg of mass gained per kcal ingested) at the peak of overfeeding in control (n=12) and ExpOF mice (n=8) from Fig. 1a-d. **d**) Total calorie exposure after the entire intervention (overfeeding + recovery) in mice from Fig. 1a-d. **e)** Representative H&E-stained images of liver tissue from control and ExpOF at d14 and d14+3 (n=6). Scale bar = 100 µm. **f-k)** Gene expression changes in liver (**f-h**) and eWAT (**i-k**) in control and ExpOF mice at d14 and d14+3 (n=6). Data shown as mean ± SEM with individual values plotted (b-k). P-values were calculated using unpaired two-tailed Welch´s t-test (c,d) and 2-way ANOVA using overfeeding and time as factors (f-k), */#/$; **/##/$$; ***/###/$$$ were used when $p < 0.05$; $p < 0.01$; $p < 0.001$, for overfeeding/time/interaction effects, respectively (f-k). n: Number of mice (biological replicates). Source data are provided as a Source Data file.

Supplementary Figure 2

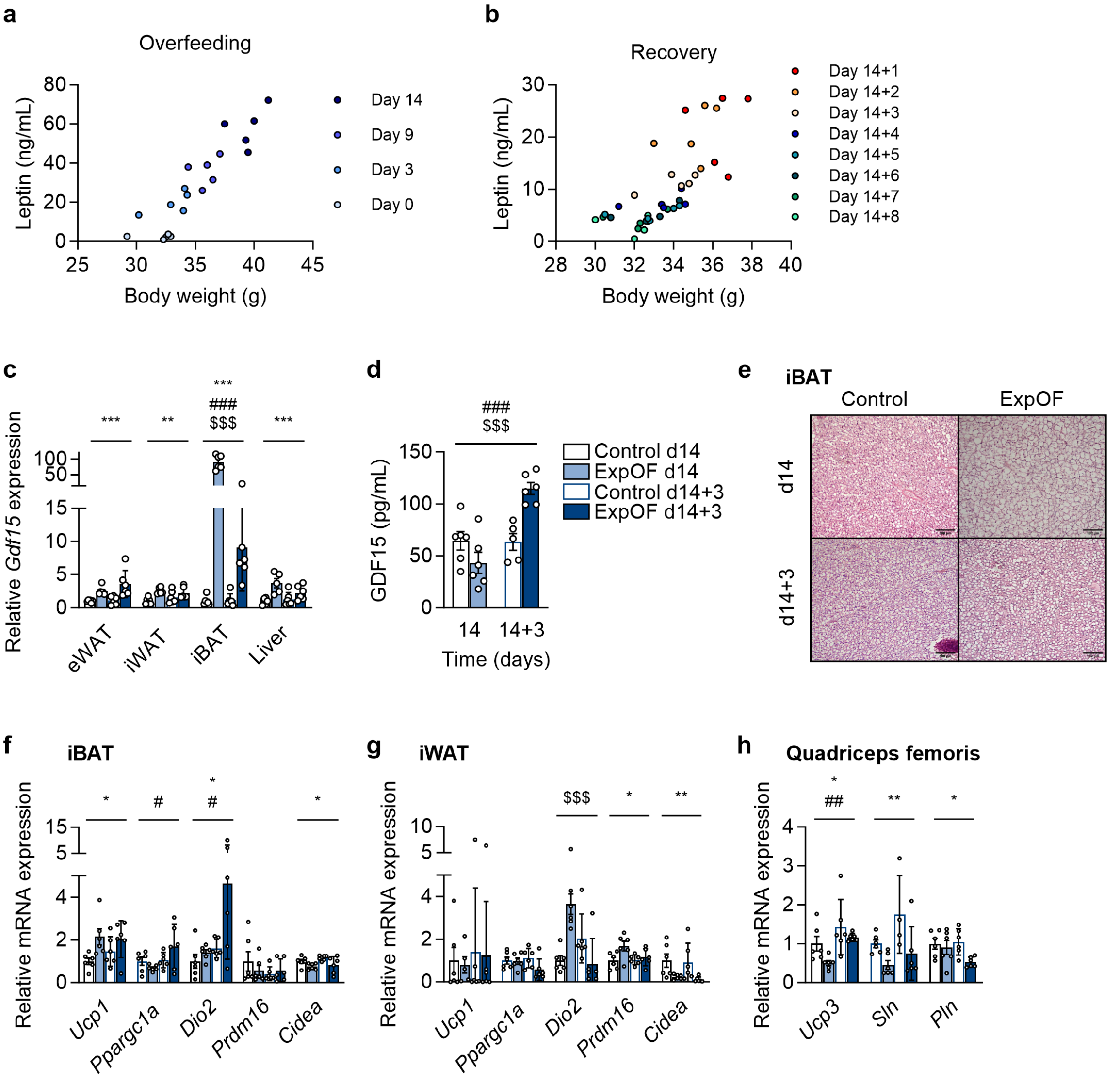

Supplementary Figure 2. Evaluation of overfeeding-induced changes in potential modulators of energy balance and their role in the response to overfeeding

a,b) Leptin levels plotted against body weight during overfeeding (**a**) and recovery (**b**) with daily resolution. Data from mice in Fig. 2a-c. **c)** *Gdf15* expression in eWAT, iWAT, iBAT, and liver in control and ExpOF mice at d14 and d14+3 (n=6). **d)** Plasma GDF15 levels (in pg/mL) in control and ExpOF mice at d14 and d14+3 (n=6). **e**) Representative images of H&E-stained interscapular brown adipose tissue (iBAT) sections. Scale bar = 100 µm. **f-h**) Thermogenic gene expression changes in iBAT (**f**), iWAT (**g**), and quadriceps femoris muscle (**h**) in control and ExpOF mice at d14 and d14+3 (n=6). The expression of *Ucp1, Ppargc1a, Dio2, Prdm16, and Cidea* (**f-g**) and *Ucp3*, *Sln*, and *Pln* (**h**) was measured and normalized to the expression of the housekeeping gene *36b4/Rplp0* and represented as relative change to the levels in control mice at d14. Data shown as mean ± SEM with individual values plotted (c,d,f,g,h). P-values were calculated using 2-way ANOVA using overfeeding and time as factors. */#/$; **/##/$$; ***/###/$$$ were used when $p < 0.05$; $p < 0.01$; $p < 0.001$, for overfeeding/time/interaction effects, respectively (c-d, f-h). n: Number of mice (biological replicates). Source data are provided as a Source Data file.

Supplementary Figure 3

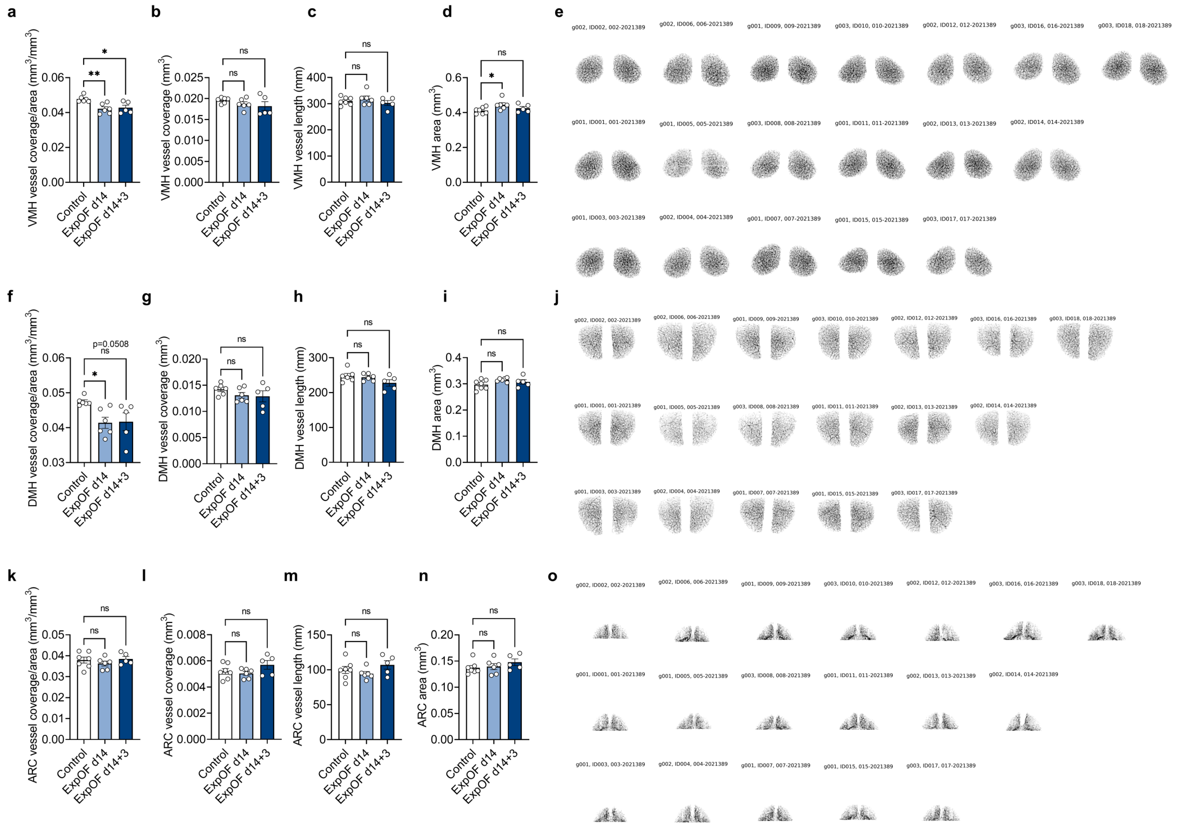

Supplementary Figure 3. Angiogenesis CD31 quantification.

a-e) VMH vessel analysis. VMH vessel coverage relative to area (**a**), total vessel coverage (**b**), total vessel length (**c**), VMH area (**d**), and pictures of analyzed areas (**e**). **f-j)** DMH vessel analysis. DMH vessel coverage relative to area (**f**), total vessel coverage (**g**), total vessel length (**h**), DMH area (**i**), and pictures of analyzed areas (**j**). **k-o)** ARC vessel analysis. ARC vessel coverage relative to area (**k**), total vessel coverage (**l**), total vessel length (**m**), ARC area (**n**), and pictures of analyzed areas (**o**). Data shown as mean ± SEM with individual values plotted (a-d, f-I, k-n). P-values were calculated using 1-way ANOVA with three groups (Control (n=7), ExpOF d14 (n=6), and ExpOF d14+3 (n=5)). */** was used when $p < 0.05$, $p < 0.01$, respectively, in post-hoc comparison after ANOVA (a-d, f-I, k-n). ns: not significant. n: Number of mice (biological replicates). Source data are provided as a Source Data file.

Supplementary Figure 4

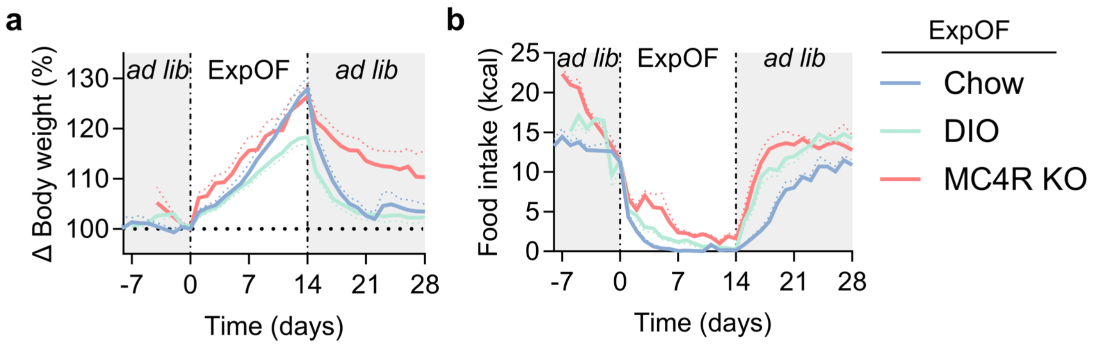

Supplementary Figure 4. Comparison of overfed mouse models

a) Comparison of body weight trajectories (changes in percentage over baseline) in overfed lean, DIO and MC4R KO mice. **b**) Daily voluntary food intake (in kcal) of mice in a during the overfeeding and recovery phases. Dashed lines represent SEM. Source data are provided as a Source Data file.
