# Supplementary material for: Protection against overfeeding-induced weight gain is preserved in obesity but does not require FGF21 or MC4R
Source: Nat Commun. 2024 Feb 8;15:1192. doi: 10.1038/s41467-024-45223-0 (PMC10853283; doi:10.1038/s41467-024-45223-0)
Supplement: Supplementary file 3 — Description of Additional Supplementary Files [file 41467_2024_45223_MOESM3_ESM.pdf]

Description of Additional Supplementary Files

Title: **Supplementary Data 1**

Description: Overfeeding protocols for all the studies carried out in this manuscript, and macronutrient composition of liquid diet used for infusion and chow diet for ad libitum feeding.

Title**: Supplementary Data 2**

Description: Targeted proteomics raw data. NPX values for the 92 proteins detected with the Mouse Exploratory panel (Olink).

Title: **Supplementary Data 3**

Description: Gene expression data from mouse hypothalamus RNA-seq comparing control and overfed mice at day 14 and day 14+3

Title: **Supplementary Data 4**

Description: Pathway enrichment data corresponding to mouse hypothalamus RNA-seq analysis comparing control and overfed mice at day 14 and day 14+3.

Title: **Supplementary Data 5**

Description: List of primers used in this study and corresponding sequences
