# Supplementary material for: Protection against overfeeding-induced weight gain is preserved in obesity but does not require FGF21 or MC4R
Source: Nat Commun. 2024 Feb 8;15:1192. doi: 10.1038/s41467-024-45223-0 (PMC10853283; doi:10.1038/s41467-024-45223-0)
Supplement: Supplementary file 9 — Reporting Summary [file 41467_2024_45223_MOESM9_ESM.pdf]

nature portfolio

Corresponding author(s): Christoffer Clemmensen

Last updated by author(s): Jan 10, 2024

Reporting Summary

Nature Portfolio wishes to improve the reproducibility of the work that we publish. This form provides structure for consistency and transparency in reporting. For further information on Nature Portfolio policies, see our Editorial Policies and the Editorial Policy Checklist.

Statistics

For all statistical analyses, confirm that the following items are present in the figure legend, table legend, main text, or Methods section.

n/a	Confirmed	
☐	☒	The exact sample size (*n*) for each experimental group/condition, given as a discrete number and unit of measurement
☐	☒	A statement on whether measurements were taken from distinct samples or whether the same sample was measured repeatedly
☐	☒	The statistical test(s) used AND whether they are one- or two-sided<br>*Only common tests should be described solely by name; describe more complex techniques in the Methods section.*
☒	☐	A description of all covariates tested
☐	☒	A description of any assumptions or corrections, such as tests of normality and adjustment for multiple comparisons
☐	☒	A full description of the statistical parameters including central tendency (e.g. means) or other basic estimates (e.g. regression coefficient) AND variation (e.g. standard deviation) or associated estimates of uncertainty (e.g. confidence intervals)
☐	☒	For null hypothesis testing, the test statistic (e.g. *F*, *t*, *r*) with confidence intervals, effect sizes, degrees of freedom and *P* value noted<br>*Give P values as exact values whenever suitable.*
☒	☐	For Bayesian analysis, information on the choice of priors and Markov chain Monte Carlo settings
☒	☐	For hierarchical and complex designs, identification of the appropriate level for tests and full reporting of outcomes
☒	☐	Estimates of effect sizes (e.g. Cohen's *d*, Pearson's *r*), indicating how they were calculated

Our web collection on statistics for biologists contains articles on many of the points above.

Software and code

Policy information about availability of computer code

Data collection	The data related to open field test (amount of time spent in the center square, compared to the sides, velocity and distance traveled) was obtained using Noldus EthoVision XT 17™ software (Noldus, NL). Histological images were acquired using light microscopy with the Zeiss Axio observer Colibri 7 inverted microscope, camera Axiocam 702 mono with objective plan-APOCROMAT 20x/0.8, ∞/0.17 (Na 0.55 WD 25mm), and Zen v3.0 software. Brains were imaged for iDisco using a Lavision ultramicroscope system II and MV PLAPO 2X C objective.
Data analysis	Adipocyte area of eWAT was measured using the watershed function on ImageJ v 1.52. Imaris software was used for 3D visualization of vascularization data. RNA-seq analyses were computed using R statistical software 4.2.3 as indicated in the Methods section. Olink proteomics data were analyzed using Metaboanalyst 5.0. The rest of data analysis was performed with GraphPad version 10.<br><br>For a list of all code used to analyze the data please refer to https://github.com/perslab/Lund-Ranea-Robles-2023/.

For manuscripts utilizing custom algorithms or software that are central to the research but not yet described in published literature, software must be made available to editors and reviewers. We strongly encourage code deposition in a community repository (e.g. GitHub). See the Nature Portfolio guidelines for submitting code & software for further information.

Data

Policy information about availability of data

All manuscripts must include a data availability statement. This statement should provide the following information, where applicable:

- Accession codes, unique identifiers, or web links for publicly available datasets
- A description of any restrictions on data availability
- For clinical datasets or third party data, please ensure that the statement adheres to our policy

Hypothalamic bulk RNA-seq data generated in this study have been submitted for GEO and are publicly available as of the date of publication under accession number GSE247825 [https://www.ncbi.nlm.nih.gov/geo/query/acc.cgi?acc=GSE247825]. Targeted proteomics raw data are available in the Supplementary information. Source data are provided with this paper. All other data generated in this study are provided in the Supplementary information/Source Data file. The source code used to analyze the RNA-seq data is available at https://github.com/perslab/Lund-Ranea-Robles-2023/.

Research involving human participants, their data, or biological material

Policy information about studies with human participants or human data. See also policy information about sex, gender (identity/presentation), and sexual orientation and race, ethnicity and racism.

Reporting on sex and gender	N/A
Reporting on race, ethnicity, or other socially relevant groupings	N/A
Population characteristics	N/A
Recruitment	N/A
Ethics oversight	N/A

Note that full information on the approval of the study protocol must also be provided in the manuscript.

Field-specific reporting

Please select the one below that is the best fit for your research. If you are not sure, read the appropriate sections before making your selection.

[x] Life sciences [ ] Behavioural & social sciences [ ] Ecological, evolutionary & environmental sciences

For a reference copy of the document with all sections, see nature.com/documents/nr-reporting-summary-flat.pdf

Life sciences study design

All studies must disclose on these points even when the disclosure is negative.

Sample size

The sample sizes were determined to be adequate based on a comprehensive review of the literature, including analogous experiments by Ravussin et al. (Cell Metabolism 2018). Additionally, data obtained from pilot experiments to optimize the overfeeding protocol, available space within the animal facility, and availability of infusion pumps were taken into account. No statistical methods were applied to determine sample size.

Data exclusions

Exclusion criteria in overfeeding studies were: 1) Clogging of cathether during experiment that was beyond repair --> Exclusion of that mouse from the study. 2) Technical issues in diet infusion that could be repaired --> Exclusion of energy intake data on that specific day.

- Figure 1d: One body weight data point on day 17 in a control mouse is excluded because that mouse was inadvertently not measured on that day
- Figure 1m: Data excluded for energy intake on specific days correspond to days where there were issues with the infusion system (usually clogging of the infusion tube) and therefore it is not possible to know how much volume went into the stomach.
- In Study 7, one MC4R KO mouse was excluded before the beginning of the study due to its low body weight (26 g).
- Figure 2b and 2c: Missing /excluded data points correspond either to samples where there was no more plasma available to perform the measurement or samples in which the levels of the hormone were below the detection level of the assay.
- Figure 2d: Missing/excluded data points correspond to samples that were below the detection limit of the assay
- Figure 2e: One data point for Fgf21 expression in iBAT on day 14+3 in ExpOF group was excluded because it was a significant outlier
- Figure 2h: Data excluded for body weight on specific days during overfeeding correspond to days where there were issues with the infusion system (usually clogging of the infusion tube). Some data points are missing in control mice during recovery because body weight was not measured on those days.
- Figure 2j: Data excluded for energy intake on specific days correspond to days where there were issues with the infusion system (usually clogging of the infusion tube) and therefore it is not possible to know how much volume went into the stomach.
- RNA-seq (Figure 3a-d): One sample in control d14 group did not pass quality control (QC) check and was excluded from analysis
- Figure 3g and 3h, and Supplementary Figure 3a-3c, 3f-3h: One sample was excluded for DMH and VMH measurements, because the signal-to-noise ratio was too high due to antibody penetration issues. Thus, the counts were only half the size as all the other measurements. It was

concluded that the algorithm could not detect the vessels in the image. ARC is close to the edge of the brain and therefore this sample had a better signal than the other brain regions.
- Figure 3k: Data excluded for body weight on specific days during overfeeding correspond to days where there were issues with the infusion system (usually clogging of the infusion tube).
- Figure 3m: Data excluded for energy intake on specific days correspond to days where there were issues with the infusion system (usually clogging of the infusion tube) and therefore it is not possible to know how much volume went into the stomach. 3 mice in the MC4R KO overfed group underwent 13 days of overfeeding instead of 14, so one value is missing on those ones to fit the peak of weight with the other mice.
- Figure 3p: Ghrelin and leptin values are missing in one ExpOF sample on day14 because blood was not collected that day on that specific mouse (tube clogged and thus will confound measurements).
- Supplementary Figure 1 Q-PCR data: Excluded data correspond to significant outliers.

Replication

Results of study 1 have been replicated in study 2, 3, 4, and 5, and in other experiments performed in the lab that are not part of this manuscript. Similar findings regarding body weight and food intake data has been replicated in other studies (like in Ravussin et al. 2018).

Results corresponding to ghrelin levels in plasma shown in Figure 2g are replicated in Figure 2k using different methodologies.

Results from study 6 were obtained in independent sets of mice, with very similar results across the different sets. We did not perform a study to replicate findings in study 7, and 8.

References:
- Ravussin Y, Edwin E, Gallop M, Xu L, Bartolomé A, Kraakman MJ, LeDuc CA, Ferrante AW Jr. Evidence for a Non-leptin System that Defends against Weight Gain in Overfeeding. Cell Metab. 2018 Aug 7;28(2):289-299.e5. doi: 10.1016/j.cmet.2018.05.029. Epub 2018 Jun 21. PMID: 29937378; PMCID: PMC6082718.

Randomization

In animal studies, both control and overfed (or mutant) mice were randomly assigned to the experimental groups, which were matched for body weight and baseline food intake. Details about experimental groups and randomization are specified in the Methods section

Blinding

Blinding was implemented whenever feasible, especially in image analysis (histology and iDisco staining). However, this practice was not applicable in overfeeding experiments where the water-infused and the diet-infused group where clearly distinguished by the experimenter. These experiments were conducted according to standardized protocols and procedures.

Reporting for specific materials, systems and methods

We require information from authors about some types of materials, experimental systems and methods used in many studies. Here, indicate whether each material, system or method listed is relevant to your study. If you are not sure if a list item applies to your research, read the appropriate section before selecting a response.

Materials & experimental systems

n/a	Involved in the study	
	x	Antibodies
x		Eukaryotic cell lines
x		Palaeontology and archaeology
	x	Animals and other organisms
x		Clinical data
x		Dual use research of concern
x		Plants

Methods

n/a	Involved in the study	
x		ChIP-seq
x		Flow cytometry
x		MRI-based neuroimaging

Antibodies

Antibodies used

CD31 (Goat anti-CD31, R&D systems cat# AF3628, lot # YZU0120101), secondary antibody (AF790 donkey a-goat lot #154504)

Validation

CD31/PECAM-1 is a classical vessel marker for mouse and human blood vessels. described in many publications for use of immuno fluorescense. Not internally validated by R&D systems but used in similar applications in many high-impact articles.
https://www.nature.com/articles/s41467-017-02158-z, https://dx.plos.org/10.1371/journal.pone.0115005, https://pubmed.ncbi.nlm.nih.gov/30498224, https://dx.plos.org/10.1371/journal.pone.0115005

Animals and other research organisms

Policy information about studies involving animals; ARRIVE guidelines recommended for reporting animal research, and Sex and Gender in Research

Laboratory animals

Wild type (WT) male C57BL/6J (Janvier, FR) mice, melanocortin 4 receptor (MC4R) knockout mice (The Jackson Laboratory, stock no. 032518, Mc4rtm1Lowl), and fibroblast growth factor 21 (FGF21) knockout mice (Fgf21tm1.1Djm, The Jackson Laboratory, stock no

033846, available at the CBMR) were used. All experiments were done at 22°C with a 12:12 h light-dark cycle (6am-6pm). Mice had ad libitum access to water and chow diet (SAFE D30, Safe Diets, France), or when indicated to high fat diet (HFD) (D12331, Research Diets, USA). All mice were single housed after surgery and during experimental overfeeding and recovery.
tion.

Study 1: Effect of experimental overfeeding on body weight and food intake
18-week-old, chow-fed male C57BL6/J mice (n=5 control; n=3 ExpOF) were overfed as described (Supplementary Data 1). Mice were observed after ExpOF to evaluate the changes in body weight and food intake until they stabilized. No tissues were collected following this study.

Study 2: Effect of experimental overfeeding on circulating factors
32-week-old chow-fed male C57BL6/J mice (n=5 control; n=5 ExpOF) overfed as described (Supplementary Data 1). Blood (20 µL) was collected, and glucose (with a glucometer), insulin, and leptin were measured on day 0, 3, 9, and 14 during the overfeeding period, and daily between day 15-22 in the recovery period.
Study 3: Experimental overfeeding of WT C57BL/6J mice: Tissue collection 1
18-week-old chow-fed male C57BL6/J mice (n=7 control; n=6 ExpOF) were overfed as described (Supplementary Data 1). On day 14, 3 control and 3 ExpOF mice were sacrificed to collect plasma, eWAT, iWAT, iBAT, liver, muscle, and brain. On day 17 (d14+3) 4 control and 3 ExpOF mice were sacrificed, and the same tissues were collected.

Study 4: Experimental overfeeding of WT C57BL/6J mice: Tissue collection 2
Study 3 was repeated to supplement tissues for analysis (day 14: n=3 control, n=3 ExpOF; day 17: n=3 control; n=3 ExpOF). (Supplementary Data 1)
Study 5: Experimental overfeeding of WT C57BL/6J mice: Collection of brains for iDISCO
18-week-old chow-fed male C57BL6/J mice (n=8 control; n=11 ExpOF) were overfed as described (Supplementary Data 1). On day of sacrifice (day 14 and 14+3), mice were anesthetized using 4% isoflurane and perfused intracardially for iDISCO staining. The study was performed twice and groups were pooled. The control groups at d14 and d14+3 were pooled (control n=8, ExpOF d14 n=6, ExpOF d14+3 n=5).

Study 6: Experimental overfeeding of DIO C57BL/6J mice – Observation of recovery after overfeeding
31-week-old obese male C57BL/6J mice (n=12 control; n=11 ExpOF) that were switched to a HFD (D12331, Research Diets, USA, 58 % energy from fat and sucrose, of which 35.4 % from fat) at 8 weeks of age were overfed as described (Supplementary Data 1). DIO mice were matched for baseline body weight (control = 43.3 ± 5.7 g, ExpOF = 43.4 ± 4.9 g) and baseline food intake (control = 15.9 ± 1.7 kcal/d, ExpOF = 16.1 ± 1.4 kcal/d) before the start of the infusions.
Study 7: Experimental overfeeding of MC4R KO mice – Observation of recovery after overfeeding, measurement of circulating hormones
17 to 21-week-old chow-fed male MC4R KO mice on a C57BL/6J background were overfed as described (Supplementary Data 1). One mouse was excluded before the beginning of the study due to its low body weight (26 g). Mice were divided into experimental groups: control (n=6) and ExpOF (n=5) matched for baseline body weight (control = 43.5 ± 3.0 g, ExpOF = 41.3 ± 1.7 g) and baseline daily food intake (control = 19.5 ± 1.2 kcal, ExpOF = 20.4 ± 1.3 kcal).

Study 8: Experimental overfeeding of FGF21 KO mice – Observation of recovery after overfeeding
20 to 30-week-old chow-fed male FGF21 KO mice on a C57BL/6J background were overfed as described (Supplementary Data 1). FGF21 KO mice were divided into experimental groups: Control (n=4) and ExpOF (n=5) matched for baseline body weight (control = 30.6 ± 2.7 g, ExpOF = 29.1 ± 3.3 g), but not for baseline daily food intake (control = 15.4 ± 1.2 kcal, ExpOF = 12.4 ± 0.5 kcal), as mice used as control were slightly older than the used for overfeeding. Energy infusions were calculated using baseline intake of mice in ExpOF group. We employed a slightly modified diet without sucrose supplementation (1.5 kcal/mL). Therefore, the macronutrient composition (35% E from fat, 50% E from carbohydrates, 15% E from protein) was slightly different to the composition of the diet used in the rest of the experiments.

Wild animals	The study did not involve wild animals
Reporting on sex	All animal studies were conducted using male mice
Field-collected samples	The study did not involve field-collected samples
Ethics oversight	All in vivo experiments were conducted according to international principles of animal care and under the approval of the Danish Ethical Committee for Animal Research and Danish Animal Experimentation Inspectorate (2018-15-0201-01457).

Note that full information on the approval of the study protocol must also be provided in the manuscript.

Plants

Seed stocks	*Report on the source of all seed stocks or other plant material used. If applicable, state the seed stock centre and catalogue number. If plant specimens were collected from the field, describe the collection location, date and sampling procedures.*
Novel plant genotypes	*Describe the methods by which all novel plant genotypes were produced. This includes those generated by transgenic approaches, gene editing, chemical/radiation-based mutagenesis and hybridization. For transgenic lines, describe the transformation method, the number of independent lines analyzed and the generation upon which experiments were performed. For gene-edited lines, describe the editor used, the endogenous sequence targeted for editing, the targeting guide RNA sequence (if applicable) and how the editor was applied.*
Authentication	*Describe any authentication procedures for each seed stock used or novel genotype generated. Describe any experiments used to assess the effect of a mutation and, where applicable, how potential secondary effects (e.g. second site T-DNA insertions, mosiacism, off-target gene editing) were examined.*
